# Prevalence and impact of ECMO cannula colonization: a single center study

**DOI:** 10.1038/s41598-025-00384-w

**Published:** 2025-05-10

**Authors:** Klaus-Georg Kreitmeier, Tobias Wertheimer, Alois Philipp, Maik Foltan, Robert Heyd, Dirk Lunz, Johannes Steinmann, Roland Schneckenpointner, Thomas Müller, Matthias Lubnow

**Affiliations:** 1 Department of Internal Medicine III/Hematology and Oncology, University Medical Center Regensburg, Regensburg, Germany; 2 Department of Internal Medicine I/Hematology and Oncology, University Medical Center Freiburg, Freiburg, Germany; 3 Department of Cardiothoracic Surgery, University Medical Center Regensburg, Regensburg, Germany; 4 Institute of Clinical Microbiology and Hygiene, University Medical Center Regensburg, Regensburg, Germany; 5 Department of Anesthesiology, University Medical Center Regensburg, Regensburg, Germany; 6 Department of Internal Medicine II/Cardiology and Pneumology, University Medical Center Regensburg, Regensburg, Germany

**Keywords:** ECMO, Cannula colonization, Biofilm, Sonication, Bloodstream infection, Outcomes research, Clinical microbiology, Infectious-disease diagnostics, Biomarkers, Cardiac device therapy

## Abstract

During ECMO therapy, infections are common and have substantial impact on treatment outcomes. However, data on the prevalence and clinical impact of microbial ECMO cannula colonization are scarce. Between October 2020 and May 2022, we evaluated 112 ECMO cannulas from 58 patients for microbial colonization using sonication fluid culture & 16S-rRNA-PCR (n = 105), or roll plate method (n = 7). 38 (33.9%) cannulas from 30 (51.7%) patients showed evidence for bacterial colonization with the predominance of coagulase-negative staphylococci (67.4%). Antipseudomonal β-Lactam treatment at cannulation and ECMO initiation at referring hospitals were associated with cannula colonization upon multivariable per-patient analysis. The prevalence of bloodstream infections was equally low and the occurrence of fever after and number of anti-infective drugs given during ECMO therapy was similar in patients with and without exposure to colonized cannulas. Finally, there was no association of cannula colonization with adverse outcomes. In summary, our data suggest that bacterial ECMO cannula colonization does not affect the clinical course of patients and thus discourage routine microbial assessment of cannula tips or even cannula exchange in the absence of clinical signs of cannula infection.

**Supplementary Information:**

The online version contains supplementary material available at 10.1038/s41598-025-00384-w.

## Introduction

Extracorporeal Membrane Oxygenation (ECMO) is a valuable treatment option for patients experiencing respiratory and/or circulatory failure. Infections are common during ECMO therapy, significantly impacting treatment outcomes^1^. While it is widely accepted that indwelling vascular catheters increase the risk for infectious complications^2^ and their removal is standard practice when evidence or suspicion of catheter-related infection arises, data on the prevalence and clinical impact of microbial colonization of ECMO cannulas are limited and show heterogeneous results^3–9^. To contribute deeper insights, we conducted a retrospective analysis of 58 patients whose cannulas were tested for microbial colonization.

## Methods

### Patient cohort, cannulation and cannula care

Between October 2020 and May 2022, 112 of 117 jugular or femoral cannulas from 58 patients who received ECMO support for at least 48 hours at our tertiary care center with in total 1234 ECMO days were evaluated for bacterial colonization. Dilatation but no skin incision was performed upon Seldinger cannulation which was the insertion technique used for most of the investigated cannulas (94.6%). Cannulation was performed by the institutional mobile ECMO team in case it was done outside our ECMO center. Cannulas were secured with cable ties and insertion site care was conducted aseptically every 48 hours or more frequently if indicated (e.g., in case of bleeding). Octenidinhydrochloride/pentoxyethanol (Octenisept, Schülke) was used for skin disinfection while dressing consisted of chlorhexidine-gluconate gel pads or sterile compresses fixed with adhesive fleece, respectively. No routine antibiotic prophylaxis was administered.

### Cannula tip harvest and microbial testing

Cannula tips were tested following cannula removal due to clinical improvement, death, or change of ECMO mode. Following spray-wipe-spray disinfection of skin penetration site with appropriate reaction and drying time (Softasept N, Braun), cannula-holding sutures were transected, and previously clamped and disconnected cannulas were removed under aseptic conditions. Cannula tips were truncated to 5 cm using sterile scissors and stored in sterile, liquid-tight specimen cups for immediate transfer to the local Institute of Clinical Microbiology and Hygiene. Out of 112 tested cannula tips, 105 underwent sonication with consecutive standard culture (SFC), inoculation of blood culture bottles (SF-BCB) and 16S-rRNA-PCR (SF-PCR) of sonication fluid while standard roll plate culture (RP) was performed for 7 cannulas (for details, see *supplement*).

### Clinical and laboratory data

Patient, treatment and laboratory data were obtained from a clinical information system (Metavision ICU, iMDsoft), an electronic lab report system (Lauris, NEXUS AG), medical reports and the institutional ECMO registry. Blood cultures were obtained when clinically indicated with bloodstream infection (BSI) being considered in case of common commensal growth in ≥ 2 blood culture bottles drawn from two separate sites within a 7-day infection window period or if ≥ 1 blood culture bottle showed growth of other bacteria^10^. Body temperature was assessed using bladder and/or tympanic measurement with the higher value being reported in case of divergence. Immunosuppressive medication was defined as corticosteroid treatment exceeding 7.5 mg prednisolone equivalent per day and/or use of other immunosuppressants during or within 7 days before the start of EMCO therapy. Major bleeding events were specified as bleeding necessitating surgical intervention or PRBC transfusions beyond routine needs (i.e., ≥ WHO grade 3^11^) with exclusion of events likely causing significant bleeding under circumstances of unaffected hemostasis (e.g., myocardial perforation). Thrombotic events within the extracorporeal circuit (ECC) were defined as visual detection of thrombi/fibrin deposits within the membrane oxygenator (MO)/centrifugal pump/ECMO cannulas, or by reports of elevated trans-oxygenator pressure gradient or reduced gas exchange capability unresponsive to air flushing. Cannulation problems were considered with report of difficult vascular puncture, high peri-interventional blood loss, excessive material consumption, organ/vessel perforation, and/or prolonged bleeding from the insertion site. Clinical signs of cannula infection were assessed upon cannula removal and considered in case of redness, and/or (non-)purulent discharge at the skin penetration site^12^.

### Data reporting, visualization and statistical analysis

Visualization and statistical analyses were conducted using GraphPad Prism 9.5.0 (GraphPad Software, Boston). Groupwise comparisons were performed using unpaired Student’s t-tests for normally distributed data and Mann–Whitney U tests if normal distribution could not be assumed based on Shapiro–Wilk normality testing. In cases of missing values, generalized linear mixed-effects models were fitted with Holm-Šídák post-hoc testing for multiple comparisons. Contingency analyses provide two-sided 95% confidence intervals (95% CI) for odds ratios (OR) with statistical significance assessed by Fisher’s exact test. Non-redundant parameters demonstrating a significant association with cannula colonization upon univariable testing were included in multivariable regression analyses. Binomial testing with expected values set to 50% (complete randomness) was employed to evaluate the effect of antibiotic therapy appropriateness on cannula colonization and clinical events. Box and whisker plots depict median values with interquartile range (IQR, boxes) and Tukey’s inner fences (whiskers) with “ + ” signs indicating mean values. For time-to-event analyses, log-rank (Mantel–Cox) tests were used and exact p values and hazard ratios ± 95% CI are reported. Statistical significance was defined as p values < 0.05.

## Results

### Prevalence of ECMO cannula colonization and microbial spectrum

**Fig. 1:**
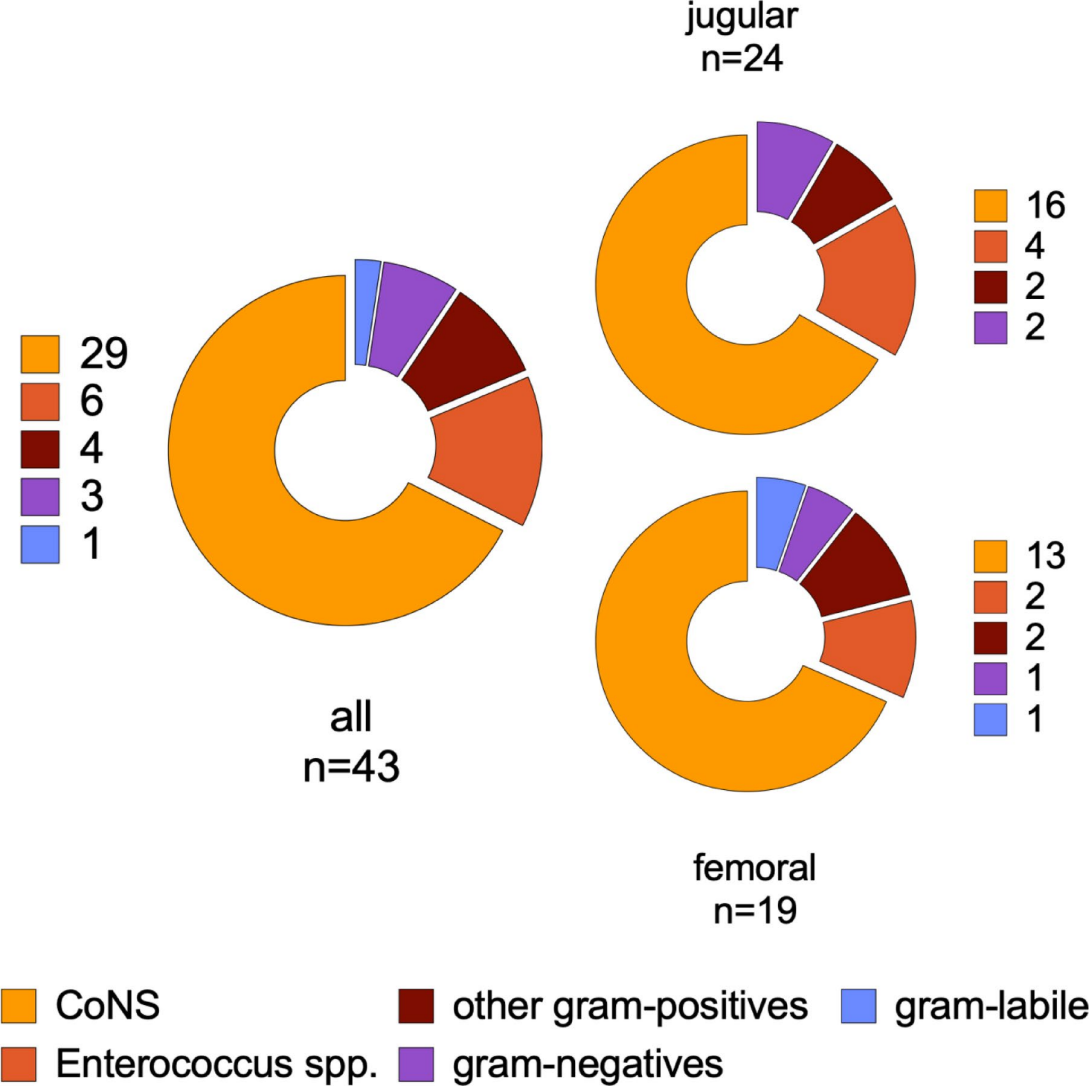
Bacteria detected in colonized cannulas. Grouping by insertion site shows similar spectrum in jugular and femoral inserted cannulas. CoNS: Coagulase-negative staphylococci.

As summarized in Table 1, bacterial colonization was evident in 38 cannulas (33.9%) from 30 patients (51.7%). 34 out of 105 sonicated cannulas (32.4%) showed colonization upon inoculation of sonication fluid in blood culture bottles, while only 7 cannulas (6.7%) yielded positive 16S-rRNA-PCR results. 13 cannulas (11.6%) showed bacterial growth either in RP cultures (2/7 cannulas in 2/4 patients) or standard SFC (11/105 cannulas in 8/54 patients). Multiple test positivity was observed in 11 cannulas (10.5% of sonicated cannulas, 9 patients). Detected bacteria were mostly coagulase-negative staphylococci (CoNS; n = 29, 67.4%), with a high proportion of oxacillin-resistant isolates (86.2%). Regarding species, S. epidermidis was predominant (55.8%), followed by Enterococcus spp. (14.0%; n = 6, including 3 VRE faecium, 2 E. faecalis, and 1 non-VRE E. faecium), other gram-positive (9.3%), and gram-negative bacteria (7.0%). Notably, 76.3% of colonized cannulas showed growth of common commensals (as defined by the CDC^10^). In 8 patients (13.8%), bacterial colonization was found in ≥ 2 cannulas. The microbial spectrum in jugular and femoral cannulas was similar (Fig. 1).

**Table 1:** Prevalence of bacterial colonization of ECMO cannula tips.

Detection method	Cannulas tested, n	Sterile, n (%)	Colonized, n (%)
Direct culture (SFC or RP culture)	112	99 (88.4)	13 (11.6)
SF in blood culture bottles (SF-BCB)	105	71 (67.6)	34 (32.4)
SF bacterial 16S-rRNA-PCR (SF-PCR)	105	98 (93.3)	7 (6.7)
≥ 1 test positive	112	74 (66.1)	38 (33.9)
≥ 2 tests positive	105	94 (89.5)	11 (10.5)
3 tests positive	105	101 (96.2)	4 (3.8)
≥ 1 test positive, excl. CoNS	112	100 (89.3)	12 (10.7)
≥ 1 test positive, excl. common commensals	112	103 (92.0)	9 (8.0)

*SFC:* sonication fluid culture, *RP:* roll plate, *CoNS:* Coagulase-negative staphylococci.

### Factors associated with ECMO cannula colonization

**Fig. 2:**
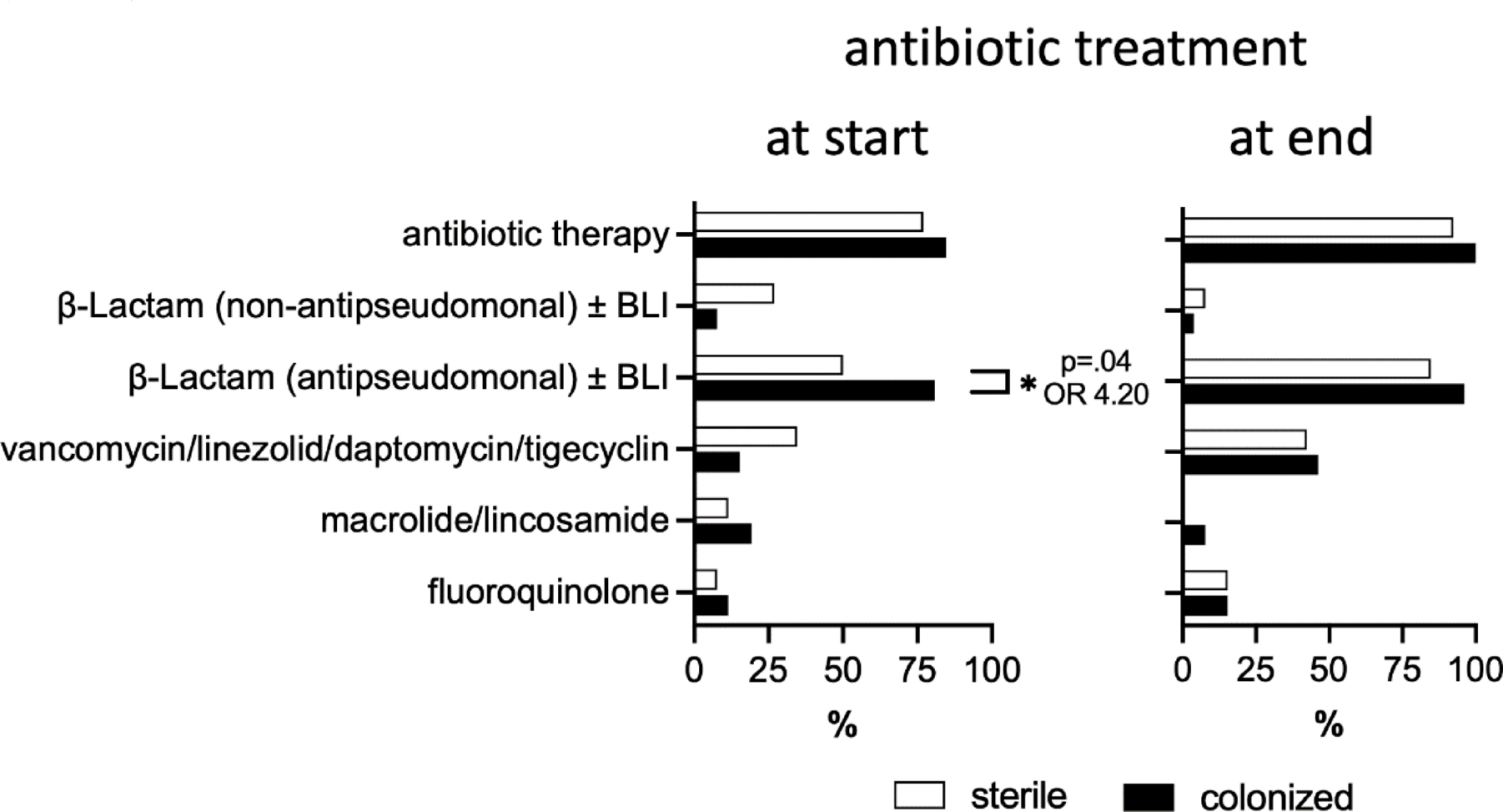
Antibiotic treatment at cannulation and decannulation (only patients with all investigated cannulas placed during the whole ECMO run included, n = 52). * indicates *p* < 0.05 upon Fisher ‘s exact test.

**Fig. 3:**
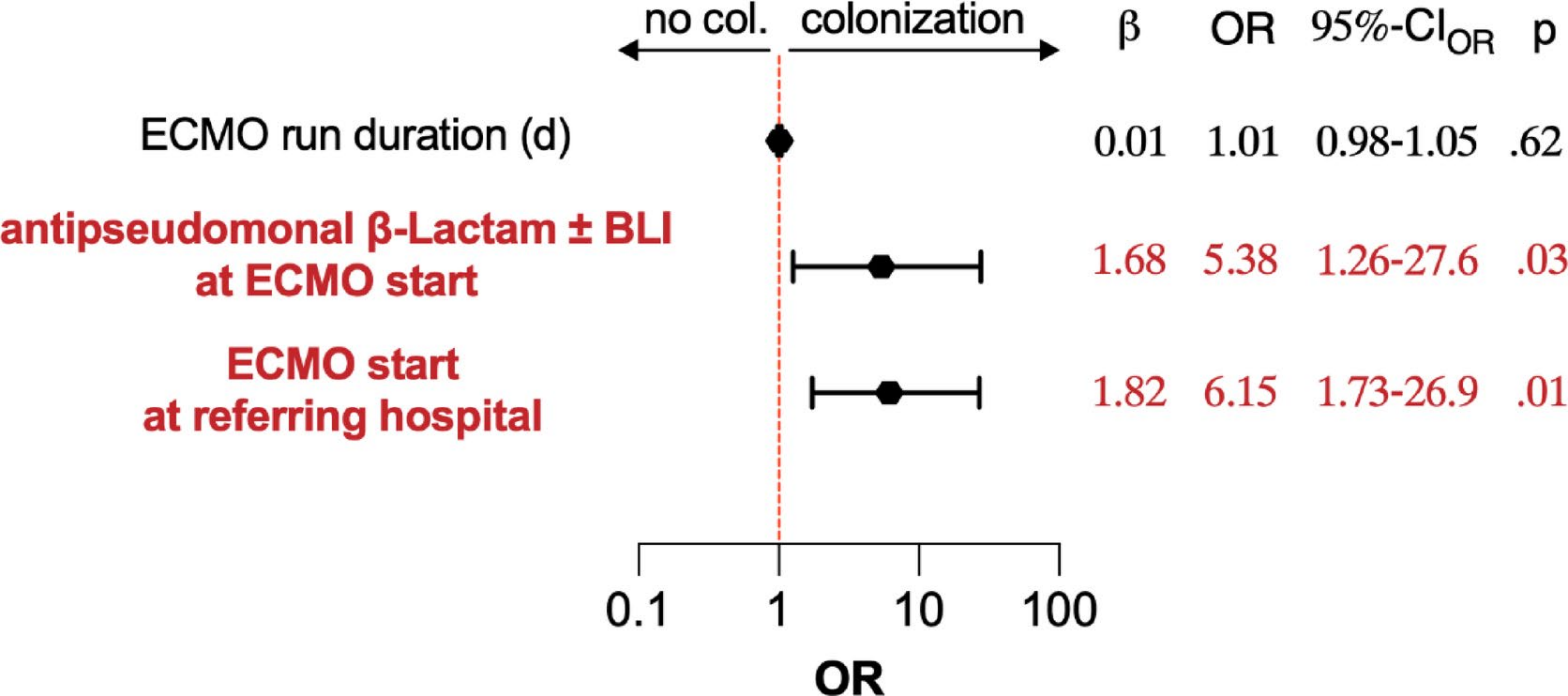
Multivariable regression analysis for factors associated with cannula colonization upon univariable per-patient analysis (only patients with all investigated cannulas placed during whole ECMO run included, n = 52).

Patient and treatment factors were analyzed for their association with cannula colonization (Table 2 and Suppl. Tables 1 and 2). De-novo cannulation using the Seldinger technique and cannulation at referring hospitals were linked to increased odds whereas cannulation at our ECMO center was associated with lower risk for cannula colonization upon univariable analysis. Position within the ECMO circuit (drainage vs. re-infusion) or the anatomical cannula insertion site (jugular vs. femoral) did not show a general association, but jugular cannulas in tracheotomized patients were more likely colonized. Patients with colonized cannulas had longer ECMO runs (mean 12 vs. 20 days, *p* = 0.02) with a likelihood ratio for colonization of 2.80 for runs lasting > 44.5 days. Interestingly, administration of antipseudomonal β‑Lactams (acylaminopenicillin + beta-lactamase inhibitor [BLI], antipseudomonal cephalosporin, or antipseudomonal carbapenem) at cannulation was associated with an increased probability for cannula colonization upon univariable analysis (Fig. 2), and colonized cannulas were more likely than random chance inserted under an antibiotic regimen not matching susceptibility of colonizing bacteria (*p* = 0.04, Suppl. Table 3). Multivariable regression (Fig. 3, per-patient analysis) confirmed association of ECMO initiation at referring hospitals and antipseudomonal β‑Lactam treatment at cannulation with cannula colonization. Multivariable per-cannula analysis is depicted in Suppl. Fig. S1.

**Table 2:** Patient characteristics and treatment data (univariable per-patient analyses).

Variable ↓ (all patients included in analyses)	All (n = 58)	Sterile (n = 28)	Colonized (n = 30)	Statistics
Age (years), median (IQR)	58.1 (52.4–62.9)	58.8 (52.6–68.1)	57.6 (51.5–60.2)	*p* = 0.24^M^
SOFA score, median (IQR)	10 (9–13)	11 (9–13)	9 (8–14)	*p* = 0.26^M^
Multiple organ failure, n (%)	17 (29.3)	8 (28.6)	9 (30.0)	OR 1.07, *p* > .99^F^ 95% CI 0.32–3.05
Body mass index (kg/m^2^), median (IQR)	27.7 (24.7–29.6)	27.5 (24.2–28.6)	27.7 (26.0–32.8)	*p* = 0.09^M^
Male gender, n (%)	45 (77.6)	21 (75.0)	24 (80.0)	OR 1.33, *p* = 0.76^F^ 95% CI 0.39–4.47
Diabetes mellitus, n (%)	17 (29.3)	7 (25.0)	10 (33.3)	OR 1.50, *p* = 0.57^F^ 95% CI 0.46–4.54
Immunosuppression, n (%)	50 (86.2)	22 (78.6)	28 (93.3)	OR 3.82, *p* = 0.14^F^ 95% CI 0.81–19.6
AB0 blood group 0, n (%)	22 (37.9)	12 (42.9)	10 (33.3)	OR 0.67, *p* = 0.59^F^ 95% CI 0.23–1.86
COVID-19 ARDS, n (%)	35 (60.3)	13 (46.4)	22 (73.3)	OR 3.17, *p* = 0.06^F^ 95% CI 1.12–8.67
CPR/cardiogenic shock/pulmonary embolism, n (%)	15 (25.9)	10 (35.7)	5 (16.7)	OR 0.36, *p* = 0.14^F^ 95% CI 0.11–1.31
Non-COVID-19 ARDS, n (%)	7 (12.1)	4 (14.3)	3 (10.0)	OR 0.67, *p* = 0.82^F^ 95% CI 0.16–2.71
Septic shock, n (%)	1 (1.7)	1 (3.6)	0 (0)	OR 0.00, *p* = 0.48^F^ 95% CI 0.00–8.40
V-V, n (%)	38 (65.5)	16 (57.1)	22 (73.3)	OR 2.06, *p* = 0.27^F^ 95% CI 0.70–5.66^F^
V-A, n (%)	14 (24.1)	10 (35.7)	4 (13.3)	OR 0.28, *p* = 0.07^F^ 95% CI 0.09–0.96
V-AV or multiple, n (%)	6 (10.3)	2 (7.1)	4 (13.3)	OR 2.00, *p* = 0.67^F^ 95% CI 0.43–11.1
RRT or PLEX while on ECMO, n (%)	12 (20.7)	6 (21.4)	6 (20.0)	OR 0.92, *p* > 0.99^F^ 95% CI 0.27–3.12
Occlusive CHX dressing, n (%)	23 (39.7)	10 (35.7)	13 (43.3)	OR 1.38, *p* = 0.60^F^ 95% CI 0.50–3.99
Time in hospital until ECMO start (days), median (IQR)	7.0 (1.0–16.3)	6.5 (2–15.5)	9.5 (1–16.3)	*p* = 0.99^M^
Duration ECMO run (days), median (IQR)	**15.5 (8.0–30.3)**	**12.0 (4.3–27.8)**	**20.0 (13.0–37.0)**	***p*** ** = 0.02** ^**M**^

Cannulation scenario				
Variable ↓ (patients with all analyzed cannulas inserted on d0 of ECMO run included in analyses)	All (n = 53)	Sterile (n = 26)	Colonized (n = 27)	
During CPR, n (%)	5 (9.4)	4 (15.4)	1 (3.7)	OR 0.21, *p* = 0.19^F^ 95% CI 0.02–1.50
During *or* after CPR, n (%)	7 (13.2)	5 (19.2)	2 (7.4)	OR 0.34, *p* = 0.25^F^ 95% CI 0.06–1.92
Cannulation problems, n (%)	10 (18.9)	2 (7.7)	8 (29.6)	OR 5.05, *p* = 0.08^F^ 95% CI 0.96–25.11
Off hours (6 p.m. to 8 a.m.), n (%)	20 (37.7)	9 (34.6)	11 (40.7)	OR 1.29, *p* = 0.78^F^ 95% CI 0.42–3.90
Out of hospital, n (%)	2 (3.8)	1 (3.8)	1 (3.7)	OR 0.96, *p* > 0.99^F^ 95% CI 0.05–18.9
Referring hospital, n (%)	**28 (52.8)**	**9 (34.6)**	**19 (70.4)**	**OR 4.49,** ***p*** **= 0.01** ^**F**^ **95% CI 1.45–14.0**
ECMO center, n (%)	**23 (43.4)**	**16 (61.5)**	**7 (25.9)**	**OR 0.22,** ***p*** **= 0.01** ^**F**^ **95% CI 0.07–0.69**

*CPR:* Cardiopulmonary resuscitation, *RRT:* Renal replacement therapy, *PLEX:* Plasma exchange, *CHX:* Chlorhexidine.

^M^Mann-Whitney U test, ^F^Fisher’s exact test.

Significant values (p < 0.05) are in bold.

### Anti-infective therapy, BSI, post-ECMO fever and inflammation parameters

**Fig. 4:**
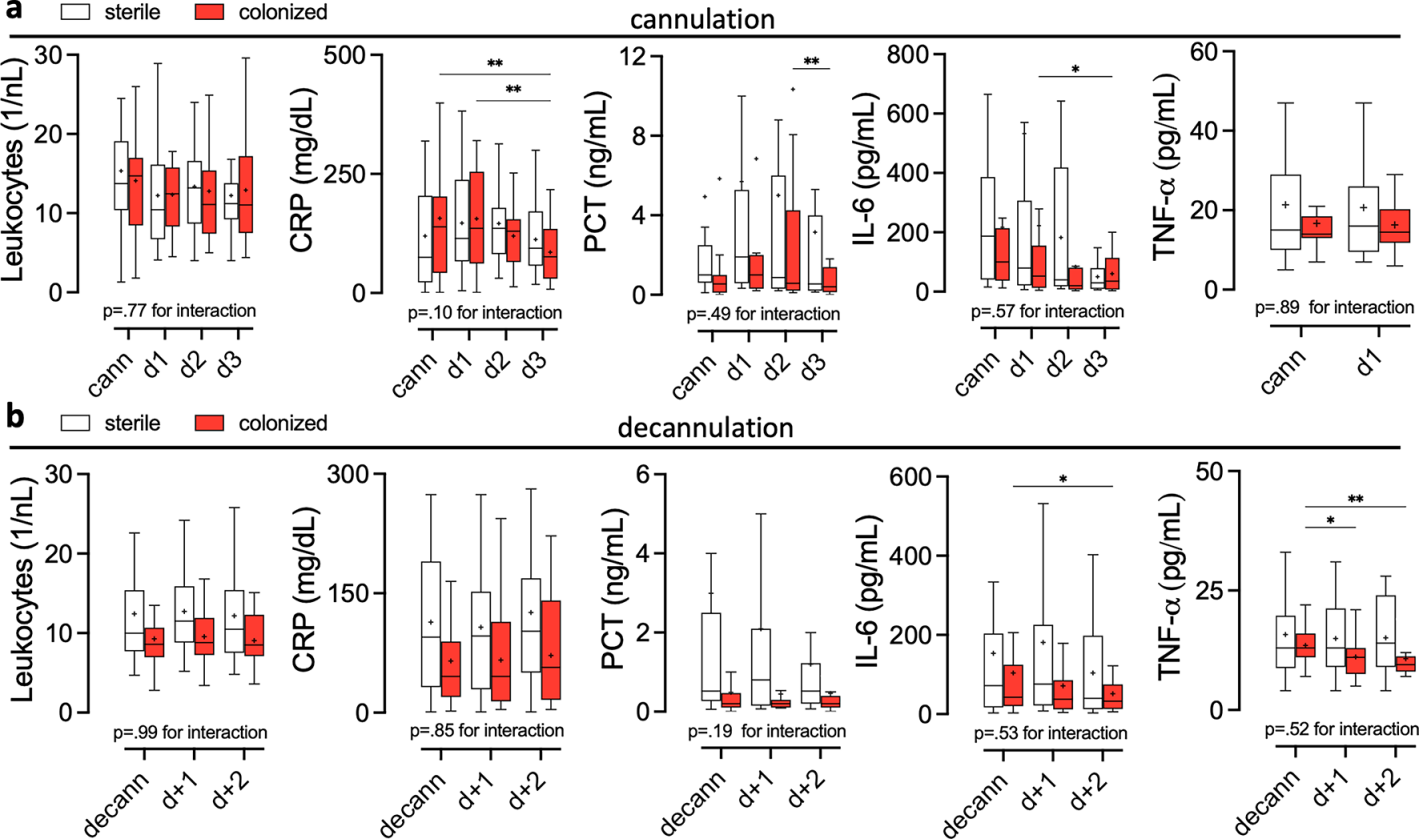
Leukocyte counts and inflammation parameters at (**a**) cannulation and (**b**) decannulation. *CRP:* C-reactive protein, *PCT*: procalcitonin, *TNF-α:* Tumor necrosis factor α, *cann:* cannulation, *decann:* decannulation. Only data from patients with all investigated cannulas placed during whole ECMO run included. *p < 0.05, **p < 0.01.

Regardless of cannula colonization status, patients received in median 3 antibiotics (*p* = 0.86) and 4 antibiotics/antimycotics (*p* = 0.39) during ECMO therapy which translated into an even lower number of antibiotic agents used per ECMO day in patients with colonized cannulas (*p* = 0.02; Suppl. Table 2). Non-deescalation modification of anti-infective therapy within 72 hours after decannulation was equally prevalent in both groups (*p* = 0.77) and there was no significant difference in the prevalence of BSI (Table 3). Microbial spectrum found in patients’ blood predominantly consisted of enterococci (Suppl. Fig. S2) and merely in one patient with BSI (the only one in the whole cohort for whom local signs of cannula infection were reported), concordance between blood culture isolates and ECMO cannula colonizing bacteria (S. epidermidis) was found. Post-ECMO fever occurred equally frequently in both groups (Table 3) and was not prevented by antibiotic treatment fitting colonizing bacteria at decannulation (Suppl. Table 3). Generally, inflammation parameters upon cannulation (Fig. 4a) and decannulation (Fig. 4b) were similar in both cohorts but IL-6 and TNF-α levels showed a significant decline in patients who had colonized cannulas removed.

**Table 3:** Occurrence of post-ECMO fever and bloodstream infections.

Outcome ↓ (patients with all investigated cannulas inserted during the whole ECMO run included in analyses)	All	Sterile	Colonized	Statistics
Fever ≥ 38 °C post ECMO until d + 3^#^	42 (85.7)	23 (88.5)	19 (82.6)	OR 0.62, *p* = 0.69^F^ 95% CI 0.14–2.57
Patients with bloodstream infection episodes > 48h on ECMO and + 24h^$^	6 (11.5)	2 (7.7)	4 (15.4)	OR 2.18, *p* = 0.67^F^ 95% CI 0.46–12.2
Patients with bloodstream infection(s) on ECMO ± 14d^$^	11 (21.2)	5 (19.2)	6 (23.1)	OR 1.26, *p* > 0.99^F^ 95% CI 0.36–4.56
Incidence of BSI episodes > 48h on ECMO and + 24h; per 10^3^ ECMO days^$^	4.9	3.9	5.5	
Incidence of BSI episodes on ECMO ± 14d per 10^3^ ECMO days^$^	9.7	11.8	8.3	

^F^Fisher’s exact test. ^#^n = 49, 3/52 patients not evaluable due to death without fever before d + 3. ^$^n = 52, patients not surviving the whole post ECMO period (+ 24 h/ + 14d) included. *BSI*: Bloodstream infection.

### Coagulation and bleeding

There was neither a significant difference in the prevalence of major bleeding events nor was there an association between cannula colonization and the occurrence of ECMO cannula-related vascular thrombosis or thrombotic events within the ECC (Suppl. Table 4). The prevalence and median time to the first MO exchange were equal. However, in the context of longer ECMO runs, patients with colonized cannulas had a significantly lower overall MO exchange frequency (71 vs. 123 MO per 10^3^ ECMO days, *p* = 0.004). Notably, when stratification was performed by colonization status of *drainage* cannulas, there was also no association of colonization with *drainage* cannula-associated venous thrombosis (OR 1.00, 95% CI 0.73–1.49, *p* > 0.99) or occurrence of thrombotic events within the extracorporeal circuit (OR 0.67, 95% CI 0.20–2.06, *p* = 0.56). Coagulation analyses revealed lower minimal vWF activity (*p* = 0.03) with almost equal nadir vWF antigen levels in patients with colonized cannulas, resulting in a trend-wise lower minimal vWF:activity/vWF:antigen ratio (Suppl. Fig. S3a) with similar peri-cannulation/decannulation kinetics (Suppl. Fig. S3b). Minimal platelet counts during ECMO therapy were equal in both groups (Suppl. Fig. S3a), with comparable frequency of platelet transfusions (Suppl. Fig. S3c) and similar kinetics after start and termination of ECMO therapy (Suppl. Fig. S3b). Patients with sterile cannulas had higher factor XIII activity at cannulation with a significant drop within the first day of ECMO therapy (Fig. S3b). While nadir FXIII activity was lower only by trend (*p* = 0.055), it showed a significant reduction at decannulation in patients with colonized cannulas but equalized thereafter (Suppl. Fig. S3a,b). There was no difference with respect to d-dimers, but fibrinogen levels declined during the first 3 days of ECMO therapy in patients who later showed cannula colonization. In the further course, nadir fibrinogen and maximum d-dimer levels were equal with similar post-decannulation kinetics (Suppl. Fig. S3a,b). Finally, the frequency of packed red blood cell (PRBC) transfusions was even lower in patients with colonized cannulas and there was significantly less use of prothrombin complex concentrates (PCC; Suppl. Fig. S3c).

### Duration of ECMO therapy, time to discharge from ICU, survival and functional outcomes

**Fig. 5:**
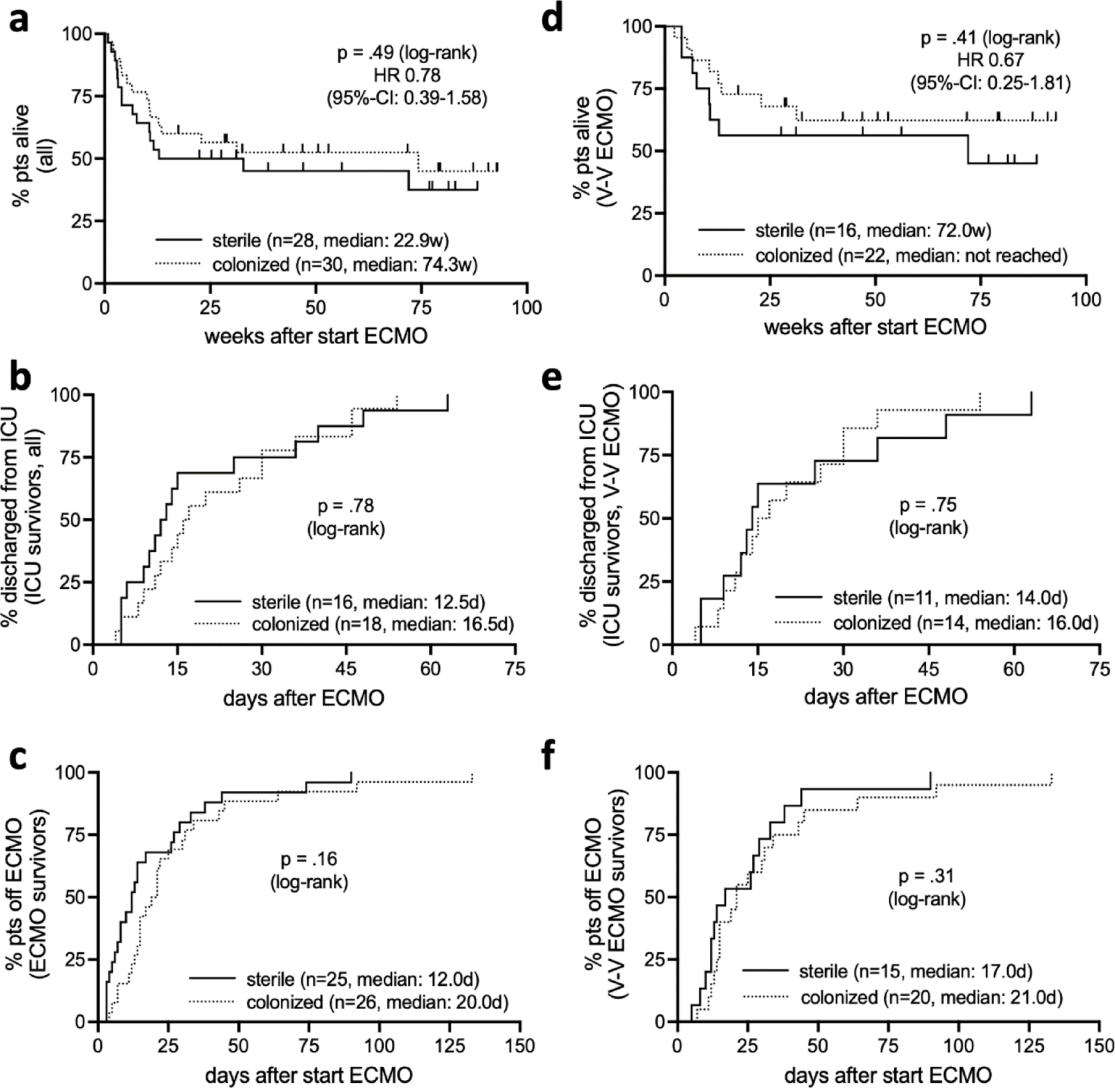
Similar overall survival (**a**, and** d** for V-V ECMO only), time do discharge from ICU (**b**, and **e** for V-V ECMO only) and time on ECMO for survivors (**c**, and **f** for V-V ECMO only) with and without cannula colonization.

Cannula colonization showed no association with adverse outcomes in terms of overall survival, time to discharge from ICU and time to ECMO weaning (Fig. 5a–c), as well as ICU mortality (Suppl. Table 5). This remains consistent after the exclusion of V-A ECMO patients (Fig. 5d–f and Suppl. Table 5). Additionally, there was no adverse effect of cannula colonization on these outcomes in subgroup analyses for cannula colonization with other than common commensal bacteria (Suppl. Fig. S4), multiple test positivity (Suppl. Fig. S5) and multiple cannula colonization (Suppl. Fig. S6). Lack of susceptibility of colonizing bacteria to the antibiotic regimen administered at decannulation did not predict worse outcomes (Suppl. Fig. S7). Finally, functional outcomes (ECOG performance status & cerebral performance category [CPC] scale score) at last contact were similar between groups in all patients and the subgroup of V-V ECMO patients, respectively (Suppl. Table 5).

## Discussion

### Prevalence, spectrum and factors associated with ECMO cannula colonization

We observed bacterial colonization in ~ 1/3 of ECMO cannulas with ~ 50% of ECMO patients being exposed to ≥ 1 colonized cannula. 16S-rRNA-PCR did not improve detection sensitivity as only 2/105 (1.9%) cannulas showed positive PCR results in the absence of cultural growth. In further 2 cases (1.9%), there was a discrepancy between PCR and culture regarding the identified species while 3/105 (2.9%) cannulas showed matching results. Additionally, multiple test positivity was not associated with worse outcomes. In the context of a long median ECMO run duration (15.5 days), cannula colonization rate was higher than previously reported by Thomas et al. (cannula colonization in 41.7% of V–V ECMO patients^5^), Yeo et al. (10.5% of patients showing cannula colonization by means of roll-plate or sonication fluid culture^7^ and 19.8% of patients with culture-positive cannula biofilm^3^), and Fuentes et al. (colonization in 26% of cannulas in context of vancomycin/daptomycin prophylaxis^6^). Interestingly, inoculation of blood culture bottles yielded substantially higher positivity rates than standard cultures which is possibly due to the larger sonication fluid volume used for the former. Notably, antibiotic treatment at decannulation did not appear to prevent diagnostic cultural growth as ~ 50% of cannulas showing bacterial colonization were harvested under an antibiotic regimen matching the susceptibility of colonizing bacteria. Although a significant proportion of patients was exposed to immunosuppressants (86.2%) or suffered from diabetes mellitus (29.3%), we did not detect cannula colonization by Candida spp. The prevalent use of antimycotic drugs (19.2% at cannulation and 53.8% at decannulation) might have prevented fungal colonization but possibly also hampered cultural detection. In line with previous findings^5–7,9^, the majority of cannula colonizing bacteria found in our study were CoNS, with an expectedly high prevalence of oxacillin-resistance^13^. Interestingly, we found an association of cannula colonization with use of broad-spectrum antipseudomonal β‑Lactams (acylaminopenicillin + BLI, antipseudomonal cephalosporin, or antipseudomonal carbapenem) at the time of initiation of ECMO therapy. We speculate that antipseudomonal β-Lactams might exert a stronger selection pressure on the skin flora than antibiotics with a narrower spectrum, resulting in a more pronounced overgrowth of resistant skin commensals (such as oxacillin-resistant CoNS), which are later found to colonize ECMO cannulas. Thereby, vancomycin, linezolid, daptomycin and tigecycline, which typically cover (oxacillin-resistant) CoNS^14,15^, seem to counterbalance this phenomenon as 12 out of 13 patients that had received these antibiotics at cannulation were simultaneously exposed to antipseudomonal β-Lactams and showed a trend towards even lower odds for cannula colonization.

However, our findings do *not* support routine antibiotic prophylaxis with these agents for the prevention of ECMO cannula colonization for multiple clear reasons: Firstly, the colonization rate in our study was only slightly higher than previously reported by others using vancomycin/daptomycin prophylaxis (notably also with predominance of CoNS^6^), suggesting limited efficacy of this approach. Secondly, the growing resistance of gram-positive bacteria towards these agents^16–19^ prohibits non-critical prophylactic use for obvious reasons. Additionally, the use of antibiotics could increase susceptibility towards invasive fungal infections^20^ with preclinical data indicating that vancomycin could be of particular relevance regarding this concern^21^. Finally and most importantly, we did not observe altered outcomes in patients with bacterial cannula colonization which indicates a lack of clinical benefit. However, our observation that antipseudomonal broad-spectrum β-Lactams may even increase the risk for cannula colonization might be relevant under other circumstances (e.g., cardiac device implantation or arthroplasty) and thus should be subject to further (prospective) evaluation.

With mainly peri-cannulation factors showing significant association with ECMO cannula colonization status, our findings suggest that cannulation itself might be the crucial event for cannula colonization. However, longer ECMO run duration (as reported previously^5^) was also associated with cannula colonization upon univariable per-patient analysis, but there was no effect upon multivariable analysis and per-cannula analysis only showed a trend towards longer indwelling time. Furthermore, there was no general association of anatomic position with cannula colonization but jugular cannulation in tracheotomized patients (tracheostomy in 16/17 cases performed after jugular cannulation) was associated with increased odds for colonization. However, markedly longer ECMO runs in these patients could be confounding (median 13 days in non-tracheotomized vs. 33 days in tracheotomized patients, *p* = 0.0001).

### ECMO cannula colonization is not associated with increased BSI frequency

BSI incidence and prevalence fell within the lower range of previous reports^22^. Interestingly, we did not observe a significant increase in BSI among patients with colonized cannulas. Only one patient who presented with clinical signs of cannula infection showed a correlation between bacterial species found in blood and cannula cultures, indicating a cannula-related BSI. Given the challenges of retrospective reporting of infection episodes in ECMO patients, we analyzed the use of anti-infective drugs as a surrogate for infection burden. We found equal total and even lower per time numbers of antibiotic agents administered in patients with colonized cannulas, which argues against clinically relevant increased or aggravated infections. To assess the potential impact of cannula biofilm stripping during removal on post-ECMO infection episodes, we evaluated prevalence of fever and non-deescalation change of anti-infective therapy after ECMO therapy and found no association with cannula colonization status. Furthermore, while we did not observe meaningful alterations in leukocyte counts, CRP and PCT levels, there was a drop in levels of upstream mediators (IL-6 and TNF-α) after decannulation in patients with colonized cannulas, possibly indicating withdrawal of an inflammatory stimulus. However, there was no evidence for any clinical impact of this finding.

### Cannula colonization does not affect key outcome parameters

We did not observe a significant effect of cannula colonization on time to discharge from ICU, which we would have expected if post-ECMO SIRS or BSI were prominent issues in patients with colonized cannulas. This finding is consistent with a previous report^5^ and remains true upon subgroup analysis as described above (see *Duration of ECMO therapy, time to discharge from ICU, survival and functional outcomes*). Furthermore, cannula colonization did not show an effect on functional outcomes and ICU mortality. There was no link between ECMO cannula colonization and overall survival, which aligns with previously reported unaltered 28-day^6^ and 90-day^5^ survival rates. On the other hand, results from studies finding worse overall survival with a higher incidence of BSI in association with cannula colonization^7^ or primarily stratifying by presence of BSI^8^ may be explained by higher BSI rates itself rather than cannula colonization. It is worth noting that a study investigating membrane oxygenator colonization reported association with higher mortality^23^, suggesting that this might be the more critical part of the ECC regarding microbial colonization.

### No association of cannula colonization with clinically relevant coagulopathy

We did not find clinical or laboratory evidence for coagulopathy in patients with colonized cannulas, which we had discussed as a potential consequence of MO colonization previously^24^. The time-dependency of the drop in vWF activity to antigen ratio during ECMO therapy^25^ could explain our findings regarding vWF activity and antigen, but we cannot exclude the contribution of a bacterial/biofilm effect. Bacterial vWF binding leading to thrombosis has been proposed as an immune evasion mechanism^26^. However, there was no evidence for a colonization-associated increase in cannula-associated vascular thrombosis or an excess of thrombotic events within the ECC. Although causation remains elusive, we speculate that lower overall MO exchange frequency in patients with colonized cannulas, who had longer ECMO runs, was rather due to a higher threshold for > 2 MO exchanges during the zenith of the COVID-19 pandemic than due to the effects of cannula colonization. Conversely, lower MO exchange frequency could be the cause rather than the effect of cannula colonization as an association of MO and cannula colonization has been described previously^23^. While maximum free hemoglobin levels were similar, all cases of pump head thrombosis occurred in systems connected to colonized cannulas. However, event rate in our study was too low to draw valid conclusions. The same applies to the prevalence of intracranial hemorrhage (ICH) with the additional constraint that in 3 out of 5 patients, ICH was detected within 24 hours after ECMO start and was thus likely related to other factors^27^. Although we found lower FXIII activity in patients with cannula colonization, there was no increase in bleeding events or transfusion requirements as it has previously been found with ECMO-associated acquired FXIII-deficiency^28^. Interestingly, a role for FXIII has been described for bacterial clearance^29^, while acquired FXIII deficiency was reported in ICU patients with COVID-19^30^ (the latter being more prevalent in the group of patients with colonized cannulas in our study). While fibrinogen levels declined after cannulation in patients with colonized cannulas, kinetics were similar between groups following decannulation. In the context of equal d-dimer levels and the simultaneous drop in CRP and IL-6 levels, the initial drop likely reflects reduced fibrinogen synthesis rather than consumption. Finally, we found an even lower frequency of PRBC and PCC transfusions in patients with colonized cannulas which further argues against clinically relevant cannula colonization-associated coagulopathy.

### Limitations

A significant proportion of patients suffered from COVID-19, which comes with potentially confounding factors including a distinct inflammatory phenotype^31^ and state of coagulopathy^32^. Despite strict adherence to disinfection and aseptic handling, we cannot exclude contamination with skin-colonizing bacteria during cannula harvest. Therefore, apparent common commensal colonization could be at least partly due to contamination. However, the fact that mainly peri-cannulation factors predicted cannula colonization strongly suggests otherwise. Due to low rates of certain events such as ICH and pump head thrombosis, this study lacks the power to detect an association between cannula colonization and these events.

## Conclusion

Our data suggest that bacterial cannula colonization does not impact the clinical course of ECMO patients and thus discourage routine testing or even the exchange of ECMO catheters in the absence of clinical signs for cannula infection.

## Electronic supplementary material

Below is the link to the electronic supplementary material.

Supplementary Material 1

## Data Availability

The datasets used and analyzed during the current study are available from the corresponding authors upon request.
