# Supplementary material for: Prevalence and impact of ECMO cannula colonization: a single center study
Source: Sci Rep. 2025 May 10;15:16278. doi: 10.1038/s41598-025-00384-w (PMC12064782; doi:10.1038/s41598-025-00384-w)
Supplement: Supplementary file 1 — Supplementary Material 1 [file 41598_2025_384_MOESM1_ESM.docx]

# **Supplement to “Prevalence and impact of ECMO cannula colonization: a single center study” by Kreitmeier et al.**

# **Cannula testing and antibiotic susceptibility evaluation**

For sonication, cannula tips were overlayed with phosphate-buffered saline (PBS) under a laminar-flow hood and sonicated at 40kHz (200W) within an ultrasound bath (BactoSonic, Bandelin) for 5 minutes. Afterwards, sonication fluid was centrifuged (4600g, 15 minutes) and supernatant was discharged leaving back a bottom layer of ≥ 3 mL. Following resuspension, inoculation of blood culture bottles (pediatric & lytic anaerobic, each ≥ 1mL; BD Bactec, BD Biosciences) and standard growth media (fluid: thioglycolate & brain-heart infusion broth; solid: Chocolate blood, fresh blood & Schaedler agar) was performed and additionally, a 0,5mL aliquot of the bottom layer was used for bacterial 16S-rRNA-PCR. All cultures were incubated under standard aerobic/anaerobic conditions and evaluated for growth up to 14 days after inoculation. Median time to positivity of sonication fluid inoculated blood culture bottles was 23.6 hours (IQR 16.9-27.9) with no significant difference between coagulase-negative staphylococci (CoNS) vs. other bacteria (p=.40). Bacterial identification was performed by MALDI-TOF MS (Bruker Daltonics, Bremen) while antibiotic susceptibility was tested using a BD Phoenix M50 system (BD, Heidelberg, Germany) with results being reported according EUCAST 8.1 & NAK until January 26, 2021 or EUCAST 11.0 & NAK thereafter. In case susceptibility testing was not feasible (e.g., due to lack of cultural growth), cases were excluded from analyses that stratified by the latter while categorization was performed with respect to susceptibility of *all* bacteria colonizing the respective cannula. For CoNS, susceptibility towards (acyl-)aminopenicillins plus beta-lactamase-inhibitor (BLI) was inferred from oxacillin susceptibility.^1^ Cannulas investigated by standard roll-plate culture were included in analyses as positivity rates were similar to sonication and as it has previously been shown that sonication fluid and roll-plate cultures yield equal sensitivity for detection of central venous catheter colonization.^2,3^

# **Anticoagulation**

Primary anticoagulation was performed with heparin (target aPTT 60 seconds) for the vast majority of patients (91.4%) with only 5 patients receiving first-line argatroban (8.6%, thereof 3 pts within the colonized cannulas cohort, target aPTT 50 seconds). During the course of ECMO therapy, switch of anticoagulant from heparin to argatroban was performed for 8 patients (4 pts with colonized cannulas) while heparin was used as second-line anticoagulant in 3 patients that primarily received argatroban (1 pt with colonized cannula).

# **ECMO systems**

Tested cannulas were from the following manufacturers: Maquet Cardiopulmonary (n=109), Getinge (Avalon; n=2) and Chalice Medical Ltd. (ParaGlide; n=1). ECMO circuits consisted of Rotaflow RF-32 (n=13), Cardiohelp (n=27, both Maquet Cardiopulmonary), Medos Deltastream DP3 (n=17, XENIOS AG) or Sorin/Dideco (n=1, Sorin Group) pumps with PLS (n=20) and Cardiohelp 5.0/7.0/IR (n=32 ,both Maquet Cardiopulmonary), Deltastream/Hilite7000LT (n=27), iLA active (n=1, both XENIOS AG), Paragon (n=13, Chalice Medical Ltd.), ECC.O5 (n=5, Sorin Group) or Nautilus (n=4, Medtronic) membrane oxygenators.

1. Bard, J. D., Hindler, J. A., Gold, H. S. & Limbago, B. Rationale for Eliminating Staphylococcus Breakpoints for β-Lactam Agents Other Than Penicillin, Oxacillin or Cefoxitin, and Ceftaroline. *Clin. Infect. Dis. Off. Publ. Infect. Dis. Soc. Am.* **58**, 1287–1296 (2014).

2. Erb, S. *et al.* Sonication for Diagnosis of Catheter-Related Infection Is Not Better Than Traditional Roll-Plate Culture: A Prospective Cohort Study With 975 Central Venous Catheters. *Clin. Infect. Dis.* **59**, 541–544 (2014).

3. Slobbe, L., el Barzouhi, A., Boersma, E. & Rijnders, B. J. A. Comparison of the Roll Plate Method to the Sonication Method To Diagnose Catheter Colonization and Bacteremia in Patients with Long-Term Tunnelled Catheters: a Randomized Prospective Study. *J. Clin. Microbiol.* **47**, 885–888 (2009).

# **Supplemental tables**

| **Variable ↓** | All  (n=112) | Sterile  (n=74) | Colonized  (n=38) | statistics |
| --- | --- | --- | --- | --- |
| Jugular, n (%) | 43 (38.4) | 24 (32.4) | 19 (50.0) | OR 2.08, p=.10^F^  95% CI: 0.90-4.47 |
| **Jugular in tracheotomized pts, n (%)** | **16 (15.2)** | **6 (8.1)** | **11 (28.9)** | **OR 4.62, p=.01^F^**  **95% CI: 1.66-14.1** |
| Femoral | 69 (61.6) | 50 (67.6) | 19 (50.0) | OR 0.48, p=.10^F^  95% CI: 0.22-1.11 |
| Femoral in pts with BMI > 31.95 kg/m^2^ | 14 (12.5) | 6 (8.1) | 8 (21.1) | OR 3.02, p=.07^F^  95% CI: 0.98-9.89 |
| Venous, n (%) | 95 (84.8) | 62 (83.8) | 33 (86.8) | OR 1.28, p=.79^F^  95% CI: 0.45-3.49 |
| Arterial, n (%) | 17 (15.2) | 12 (16.2) | 5 (13.2) | OR 0.78, p=.79^F^  95% CI: 0.29-2.20 |
| Drainage excl. dual lumen, n (%) | 52 (46.4) | 38 (51.4) | 14 (36.8) | OR 0.55, p=.17^F^  95% CI: 0.25-1.24 |
| Drainage incl. dual lumen, n (%) | 55 (49.1) | 40 (54.1) | 15 (39.5) | OR 0.55, p=.17^F^  95% CI: 0.26-1.24 |
| Dual lumen, n (%) | 3 (2.7) | 2 (2.7) | 1 (2.6) | OR 0.97, p>.99^F^  95% CI: 0.07-8.58 |
| Re-infusion excl. dual lumen, n (%) | 57 (50.9) | 34 (46.0) | 23 (60.5) | OR 1.80, p=.17^F^  95% CI: 0.81-3.92 |
| Cannulation out of hospital, n (%) | 6 (5.4) | 4 (5.4) | 2 (5.3) | OR 0.97, p>.99^F^  95% CI: 0.18-4.34 |
| **Cannulation at referring hospital, n (%)** | **56 (50.0)** | **30 (40.5)** | **26 (68.4)** | **OR 3.18, p=.01^F^**  **95% CI: 1.37-6.87** |
| **Cannulation at ECMO center, n (%)** | **50 (44.6)** | **40 (54.1)** | **10 (26.3)** | **OR 0.30, p=.01^F^**  **95% CI: 0.13-0.73** |
| **Seldinger de novo, n (%)** | **91 (81.2)** | **56 (75.7)** | **35 (92.1)** | **OR 3.75, p=.04^F^**  **95% CI: 2.03-11.1** |
| Seldinger over catheter, n (%) | 15 (13.4) | 13 (17.6) | 2 (5.3) | OR 0.26, p=.08^F^  95% CI: 0.06-1.14 |
| Surgical, n (%) | 6 (5.4) | 5 (6.8) | 1 (2.6) | OR 0.37, p=.66^F^  95% CI: 0.03-2.95 |
| Cannulation during CPR, n (%) | 14 (12.5) | 11 (14.9) | 3 (7.9) | OR 0.49, p=.38^F^  95% CI: 0.14-1.82 |
| Cannulation during or after CPR, n (%) | 20 (17.9) | 16 (21.6) | 4 (10.5) | OR 0.43, p=.20^F^  95% CI: 0.15-1.37 |
| Cannula-ass. thrombosis (art. & ven.), n (%)^#^ | 25 (28.1) | 16 (28.1) | 9 (28.1) | OR 1.00, p>.99^F^  95% CI: 0.73-1.49 |
| Venous cannula-ass. thrombosis, n (%)^#^ | 24 (27.0) | 15 (26.3) | 9 (28.1) | OR 1.03, p>.99^F^  95% CI: 0.43-2.83 |
| Antibiotic treatment @ cannulation, n (%) | 90 (80.4) | 58 (78.4) | 32 (84.2) | OR 1.47, p=.62^F^  95% CI: 0.55-4.15 |
| Non-antipseudomonal β-Lactam ± BLI, n (%) | 21 (18.8) | 17 (23.0) | 4 (10.5) | OR 0.40, p=.13^F^  95% CI: 0.14-1.24 |
| **Antipseudomonal β-Lactam ± BLI, n (%)** | **72 (64.3)** | **42 (56.8)** | **30 (78.9)** | **OR 2.86, p=.02^F^**  **95% CI: 1.21-6.89** |
| Vancomycin/Linezolid/Daptomycin/Tigecyclin, n (%)^$^ | 28 (25.0) | 22 (29.7) | 6 (15.8) | OR 0.44, p=.17^F^  95% CI: 0.16-1.23 |
| Macrolide/Lincosamide, n (%) | 17 (15.2) | 12 (16.2) | 5 (13.1) | OR 0.78, p=.79^F^  95% CI: 0.29-2.20 |
| Fluoroquinolone, n (%) | 10 (8.9) | 6 (8.1) | 4 (10.5) | OR 1.33, p=.73^F^  95% CI: 0.40-4.79 |
| Time in hospital until cannulation (days),  median (IQR) | 7.0 (1.0-15.0) | 6.5 (1.8-15.0) | 10.5 (1.0-16.3) | p=.47^M^ |
| Duration of cannulation (days), median (IQR) | 15.0 (8.0-30.0) | 14.0 (7.0-28.3) | 17.0 (12.8-36.3) | p=.08^M^ |

**Supplemental Table 1:** Cannulation and treatment factors (univariable *per-cannula* analysis). ^M^Mann-Whitney U test, ^F^Fisher’s exact test. ^#^23 cannulas not evaluable due to lack of vascular imaging. ^$^in 26/28 cases combined with antipseudomonal β-Lactam ± beta-lactamase inhibitor (BLI). BMI: body mass index, CPR: cardiopulmonary resuscitation.

| **Variable ↓**  (Pts with all investigated cannulas inserted during whole ECMO run included) | all  (n=52) | sterile  (n=26) | colonized  (n=26) | statistics |
| --- | --- | --- | --- | --- |
| Antibiotic treatment @ cannulation, n (%) | 42 (80.8) | 20 (76.9) | 22 (84.6) | OR 1.65, p=.73^F^  95% CI: 0.46-5.73 |
| Non-antipseudomonal β-Lactam ± BLI, n (%) | 9 (17.3) | 7 (26.9) | 2 (7.7) | OR 0.23, p=.14^F^  95% CI: 0.04-1.05 |
| **Antipseudomonal β-Lactam ± BLI, n (%)** | **34 (65.4)** | **13 (50.0)** | **21 (80.8)** | **OR 4.20, p=.04^F^**  **95% CI: 1.18-13.44** |
| Vancomycin/Linezolid/  Daptomycin/Tigecyclin, n (%)^#^ | 13 (25.0) | 9 (34.6) | 4 (15.4) | OR 0.34, p=.20^F^  95% CI: 0.11-1.23 |
| Macrolide/Lincosamide, n (%) | 8 (15.4) | 3 (11.5) | 5 (19.2) | OR 1.83, p=.70^F^  95% CI: 0.39-7.48 |
| Fluoroquinolone, n (%) | 5 (9.6) | 2 (7.7) | 3 (11.5) | OR 1.57, p>.99^F^  95% CI: 0.30-9.36 |
| Antibiotic treatment @ decannulation, n (%) | 50 (96.2) | 24 (92.3) | 26 (100) | OR ∞, p =.49^F^  95% CI: 0.47- ∞ |
| Non-antipseudomonal β-Lactam ± BLI, n (%) | 3 (5.8) | 2 (7.7) | 1 (3.8) | OR 0.48, p=.73^F^  95% CI: 0.03-4.39 |
| Antipseudomonal β-Lactam ± BLI, n (%) | 47 (90.4) | 22 (84.6) | 25 (96.2) | OR 4.55, p=.35^F^  95% CI: 0.64-57.5 |
| Vancomycin/Linezolid/  Daptomycin/Tigecyclin, n (%) | 23 (44.2) | 11 (42.3) | 12 (46.2) | OR 1.17, p>.99^F^  95% CI: 0.39-3.58 |
| Macrolide/Lincosamide, n (%) | 2 (3.8) | 0 (0) | 2 (7.7) | OR ∞, p=.49^F^  95% CI: 0.47-∞ |
| Fluoroquinolone, n (%) | 8 (15.4) | 4 (15.4) | 4 (15.4) | OR 1.00, p>.99^F^  95% CI: 0.26-3.79 |
| Antibiotic treatment paused ≥ 24 hours during ECMO run, n (%) | 14 (26.9) | 7 (26.9) | 7 (26.9) | OR 1.00, p>.99^F^  95% CI: 0.29-3.42 |
| **Outcome ↓**  (Pts with all investigated cannulas inserted during whole ECMO run included) | all  (n=52) | sterile  (n=26) | colonized  (n=26) | statistics |
| Total number of ABTx during ECMO, median (range) | 3 (1-14) | 3 (1-14) | 3 (1-8) | p=.86^M^ |
| Total number of ABTx and antifungals during ECMO, median (range) | 4 (1-15) | 4 (1-15) | 4 (1-9) | p=.39^M^ |
| **Number of ABTx per 10^3^ ECMO days,**  **median (IQR)** | **186 (133-333)** | **250 (133-471)** | **157 (117-250)** | **p=.02^M^** |
| Number of ABTx and antifungals per 10^3^ ECMO days, median (IQR) | 250 (177-333) | 314 (174-500) | 234 (175-286) | p=.09^M^ |
| Non-deescalative change of anti-infective therapy within 72hrs after ECMO^$^ | 16 (33.3) | 9 (36.0) | 7 (30.4) | OR 0.85, p=.77^F^  95% CI: 0.38-1.86 |

**Supplemental Table 2**: Anti-infective therapy (univariable *per-patient* analysis). ^M^Mann-Whitney U test, ^F^Fisher’s exact test, ABTx: antibiotics. ^#^in 12/13 cases combined with antipseudomonal β-Lactam ± beta-lactamase inhibitor (BLI), ^$^n=48, patients with death in absence of ABTx change before +72hrs after ECMO therapy excluded.

| **Outcome ↓** | n | susceptible | not susceptible | statistics |
| --- | --- | --- | --- | --- |
| **Per cannula:** **Antibiotics at cannulation**^#^ | | | | |
| **≥ 1 test positive, n (%)** | **35** | **11 (31.4)** | **24 (68.6)** | **p = .04** |
| ≥ 2 tests positive, n (%) | 11 | 3 (27.3) | 8 (72.7) | p = .23 |
| **Per cannula:** **Antibiotics at decannulation**^#^ | | | | |
| ≥ 1 test positive, n (%) | 35 | 21 (56.5) | 14 (43.5) | p = .24 |
| ≥ 2 tests positive, n (%) | 11 | 5 (45.5) | 6 (54.5) | p > .99 |
| **Per patient: Antibiotics at decannulation**^#^  (pts with all investigated cannulas being inserted during whole ECMO run and susceptibility testing available) | | | | |
| Patients with at least one colonized cannula, n (%) | 23 | 13 (52.0) | 10 (48.0) | p = .54 |
| Patients with ≥ 2 colonized cannulas, n (%) | 8 | 5 (62.5) | 3 (37.5) | p = .51 |
| Fever ≥ 38 °C post ECMO until d+3, n (%) | 18 | 11 (61.1) | 7 (38.9) | p = .48 |

**Supplemental Table 3:** Microbial test positivity and outcomes stratified by susceptibility of cannula colonizing bacteria towards antibiotic treatment at cannulation and decannulation. ^#^3 colonized cannulas not evaluable due to lack of susceptibility testing.

| **Outcome ↓**  (all pts included in analyses) | all | sterile | colonized | statistics |
| --- | --- | --- | --- | --- |
| ICH, n (%) | 5 (8.6) | 1 (3.6) | 4 (13.3)^#^ | OR 4.15, p = .35^F^  95% CI: 0.02-1.67 |
| Major bleeding (including ICH), n (%) | 19 (32.8) | 8 (28.6) | 11 (36.7) | OR 1.45, p = .58^F^  95% CI: 0.48-4.04 |
| Major bleeding (excluding ICH), n (%) | 14 (24.1) | 7 (25.0) | 7 (23.3) | OR 0.91, p > .99^F^  95% CI: 0.28-3.00 |
| ECMO cannula-ass. vascular thrombosis, n (%)^$^ | 20 (44.4) | 9 (45.0) | 11 (44.0) | OR 0.96, p>.99^F^  95% CI: 0.32-2.91 |
| Thrombotic event within ECMO circuit,  n (%) | 26 (45.6) | 12 (42.9) | 14 (48.3) | OR 1.24, p=.79^F^  95% CI: 0.46-3.43 |
| Pumphead thrombosis, n (%) | 4 (6.9) | 0 (0) | 4 (13.3) | OR ∞, p=.11^F^  95% CI: 0.93-∞ |
| ≥ 1 MO exchange, n (%) | 27 (46.6) | 12 (42.9) | 15 (50.0) | OR 1.33, p=.61^F^  95% CI: 0.50-3.62 |
| ≥ 2 MO exchanges, n (%) | 13 (22.4) | 7 (25.0) | 6 (20.0) | OR 0.75, p=.76^F^  95% CI: 0.22-2.57 |
| Time to first MO exchange (days), median (IQR) | 8.5 (3.3-11) | 9 (3.0-16) | 9 (3-12) | p=.55^M^ |
| **MO/10^3^ ECMO days, median (IQR)** | **90.9 (66.7-161)** | **123 (79.7-250)** | **70.6 (51.2-119)** | **p=.004^M^, p=.007^ULR^** |

**Supplemental Table 4:** Major bleeding/thrombotic events and MO exchange data. ^M^Mann-Whitney U test, ^F^Fisher’s exact test, ^ULR^univariable linear regression. ^#^3 pts with ICH within 24 hours of ECMO therapy, ICH: intracranial hemorrhage, MO: membrane oxygenator), ^$^n=45, pts with post ECMO vascular imaging (sonography, contrast-enhanced CT scan).

| **Outcome ↓**  (All pts included in analyses) | all | sterile | colonized | statistics |
| --- | --- | --- | --- | --- |
| Death on ICU, all pts, n (%) | 24 (41.4) | 12 (42.9) | 12 (40.0) | OR 0.89, p > .99^F^  95% CI: 0.32-2.42 |
| Death on ICU, V-V ECMO pts, n (%)^#^ | 13 (34.2) | 6 (37.5) | 7 (31.8) | OR 0.85, p = .74^F^  95% CI: 0.36-2.06 |
| ECOG @ last contact (all alive^$^),  median (range) | 1 (0-3) | 1 (1-2) | 1 (0-3) | p = .54^M^ |
| ECOG @ last contact (V-V alive^*^),  median (range) | 1 (1-3) | 1 (1-2) | 1 (1-3) | p = .56^M^ |
| CPC @ last contact (all alive^$^),  median (range) | 1 (1-3) | 1 (1-3) | 1 (1-1) | p = .09^M^ |
| CPC @ last contact (V-V alive^*^),  median (range) | 1 (1-3) | 1 (1-3) | 1 (1-1) | p = .40^M^ |

**Supplemental Table 5:** Overview of ICU mortality and functional outcomes for all and the subgroup of V-V ECMO patients. ^M^Mann-Whitney U test, ^F^Fisher’s exact test. ICU: intensive care unit, ECOG: Eastern Cooperative Oncology Group scale, CPC: cerebral performance category scale score. ^#^n=38, ^$^n=34 and ^*^n=25.

# **Supplemental figures**

multivariable *per-cannula* analysis

**Supplemental Figure S1:** Multivariable regression analysis for factors associated with cannula colonization upon univariable *per-cannula* analysis (n=112 cannulas).

**Supplemental Figure S2:** BSI-causing bacteria in patients with and without ECMO cannula colonization during and ±14 days around ECMO therapy.

**a**

decannulation

cannulation

**b**

**c**

**Supplemental Figure S3:** (**a**) Membrane oxygenator (MO) exchange frequency and nadir vWF activity/vWF antigen & vWF activity to antigen ratio, factor VIII (FVIII) activity, platelet count, fibrinogen & factor XIII (FXIII) activity as well as maximal d-dimer and free hemoglobin (hgb) levels during ECMO therapy. (**b**) Coagulation parameters and platelet counts upon cannulation and decannulation. (**c**) Red blood cell (PRBC) & platelet transfusions, coagulation factor substitution (factor XIII (FXIII), prothrombin complex concentrate (PCC), fibrinogen) and proportional time with tranexamic acid (TxA) administration. Only patients with all investigated cannulas being inserted during whole ECMO run included (n=52). * p<0.05, ** p<0.01 and *** p<0.001.

**a**

**b**

**c**

**d**

**e**

**f**

**Supplemental Figure S4:** Subgroup analyses for patients with other than common commensal cannula colonization. Overall survival in (**a**) all and (**b**) V-V ECMO patients. Time to discharge from ICU after termination of ECMO therapy in (**c**) all and (**d**) V-V ECMO ICU survivors. Time to ECMO weaning in (**e**) all and (**f**) V-V ECMO survivors.

**a**

**b**

**c**

**d**

**e**

**f**

**Supplemental Figure S5:** Subgroup analyses for patients with at least two positive tests (direct culture/S-BCB/S-PCR) in one cannula. Overall survival in (**a**) all and (**b**) V-V ECMO patients. Time to discharge from ICU after termination of ECMO therapy in (**c**) all and (**d**) V-V ECMO ICU survivors. Time to ECMO weaning in (**e**) all and (**f**) V-V ECMO survivors.

**a**

**b**

**c**

**d**

**e**

**f**

**Supplemental Figure S6:** Subgroup analyses for patients with at least two colonized cannulas. Overall survival in (**a**) all and (**b**) V-V ECMO patients. Time to discharge from ICU after termination of ECMO therapy in (**c**) all and (**d**) V-V ECMO ICU survivors. Time to ECMO weaning in (**e**) all and (**f**) V-V ECMO survivors.

**a**

**b**

**c**

**d**

**e**

**f**

**Supplemental Figure S7:** Subgroup analyses for patients with colonized cannulas stratified by antibiotic susceptibility of cannula colonizing bacteria towards the antibiotic regimen administered at decannulation. Overall survival in (**a**) all and (**b**) V-V ECMO patients. Time to discharge from ICU after termination of ECMO therapy in (**c**) all and (**d**) V-V ECMO ICU survivors. Time to ECMO weaning in (**e**) all and (**f**) V-V ECMO survivors.
